# Enhanced Fenton-like process over Z-scheme MoO_3_ surface decorated with Fe_2_O_3_ under visible light

**DOI:** 10.1038/s41598-024-58634-2

**Published:** 2024-04-05

**Authors:** Hsien-Tse Hsu, Shao-Ying Lin, Ya-Ting Lu, Yao-Yuan Chuang, Shiow-Huey Chuang

**Affiliations:** 1 Department of Applied Chemistry, National University of Kaohsiung, Kaohsiung, 81148 Taiwan; 2 Department of Chemical and Materials Engineering, National University of Kaohsiung, Kaohsiung, 81148 Taiwan

**Keywords:** Photocatalytic degradation, Fe_2_O_3_/MoO_3_ rod, Methylene blue, Fenton-like mechanism, Z-scheme, Chemistry, Materials science

## Abstract

Photocatalysts consisting of Z-scheme heterojunctions are commonly used in wastewater treatment due to their exceptional reactivity in photocatalysis and highly efficient visible-light utilization. In this work, Fe_2_O_3_-decorated MoO_3_ rods were synthesized through a two-step method and their photodegradation of methylene blue (MB) was evaluated. The Fe_2_O_3_/MoO_3_ rods were characterized by XRD, SEM, micro-Raman, XPS, UV–Vis DRS, and PL to investigate their structural, morphological, and optical properties. The results indicate that the photodegradation efficiency of Fe_2_O_3_/MoO_3_ improved through a reduction in the gap energy and persistence of a 1D hexagonal prism structure. The degradation rate of MB was enhanced from 31.7 to 91.5% after irradiation for 180 min owing to electron–hole separation and Fenton-like process. Formation of the OH radical is a key factor in the photodegradation reaction and with the addition of H_2_O_2_ the efficiency can further improve via a Fenton-like mechanism. Furthermore, the Z-scheme mechanism concurrently delineated. The Fe_2_O_3_/MoO_3_ rod composites were also found to retain high photocatalytic efficiency after being reused five times, which may be useful for future applications.

## Introduction

Wastewater generated from textile and pulp industrial manufacturing processes often contain organic dye molecules. These “colored pollutants” not only block sunlight, thereby affecting aquatic ecosystems, but are also difficult to degrade due to their stable aromatic structures. Furthermore, these organic pollutants are well known to be poisonous and cause allergies and even cancer. Therefore, removing organic pollutants from wastewater is critical^1–4^ for the United Nations’ Sustainable Development Goals. Several methods are used to remove organic pollutants from wastewater, including physical, chemical, and biological processes. Among them, utilizing semiconductor photocatalysts to degrade organic pollutants is one of the most promising techniques, especially since it is an attractive “green chemistry” approach. In photocatalytic degradation processes, hydroxyl radicals are generated from the semiconductor surface via photonic activation, which oxidizes organic pollutants and degrades them into fragments. There are many metal oxide semiconductors that can be used for photodegradation of organic dyes, such as TiO_2_^5^, Fe_2_O_3_^6^, MnO_2_^7^, CuO^8^, WO_3_^9,10^ and MoO_3_^11–15^. Their performance as photodegraders of organic dyes usually depends on their ability to utilize sunlight, which is related to their intrinsic physical properties and extrinsic structural morphologies.

TiO_2_ is a common semiconductor used for photodegradation because of its excellent photocatalytic activity, low cost, chemical stability, and low toxicity. However, the rutile and anatase forms possess band gaps of 3.0 and 3.2 eV, respectively, which correspond to the UV light region^5,16^. Therefore, the search for a metal oxide that fully utilizes the UV–visible spectrum is ongoing. The semiconductor MoO_3_^11^ has been applied in reaction catalysts, gas sensors, solar cells, and lithium ion batteries owing to its electrochemical and optical properties. There are three crystal phases of the wide-band gap semiconductor MoO_3_: orthorhombic (α-MoO_3_), monoclinic (β-MoO_3_), and hexagonal (h-MoO_3_). While α-MoO_3_ is the most thermodynamically stable phase, both the metastable β-MoO_3_ and h-MoO_3_ phases are also being investigated due to their physicochemical properties. MoO_3_ can be synthesized by microwave, sonochemical, or hydrothermal processes. Wu et al*.*^12^ found that sonochemically prepared MoO_3_ gave a material with superior performance in azo dye degradation due to a larger surface area. Chithambararaj et al*.*^13^ prepared h-MoO_3_ and α-MoO_3_ nanocrystals and found that the degradation process was slower for α-MoO_3_. This was attributed to a reduction in electron–hole pairs in the electronic band structure and also the enhancement of h-MoO_3_ due to its 1D hexagonal prism nanostructure. Li et al.^14^ prepared h-MoO_3_ from an α-MoO_3_ precursor using NaNO_3_ and transformed h-MoO_3_ back to α-MoO_3_ nanobelts using a hydrothermal technique. They also found that h-MoO_3_ nanorods had greater catalytic activity for photodegrading methylene blue (MB) due to its reduced band gap and lower electron–hole recombination rate. Based on the literature, it is apparent that the 1D hexagonal prism structures in MoO_3_ are very important for enhancing the photodegradation ability^15^.

Many types of p–n heterojunction composite metal oxides were designed to adjust their band gap energy and hence improve applicability in solar cells, gas sensing, and photocatalysis. Charge transfer reduces the recombination rate of photogenerated charge carriers, greatly improving their reactivity. The Z-scheme mechanism can also separate charge carriers to keep a greater negative potential of electrons (e^−^) and a greater positive potential of holes (h^+^), thus providing more energy for photocatalytic reactions^17–19^. The semiconductor Fe_2_O_3_ is commonly used for photodegradations because of its narrow band gap and suitable band position. It is also used to synthesize composite materials with other metal oxide semiconductors in photocatalytic applications. For example, Fe_2_O_3_/TiO_2_ photoanodes have an increase of 40% in solar conversion efficiency with fast electron transport and a short electron lifetime^20^. Toluene sensing is greatly improved by a heterojunction between NiO and α-Fe_2_O_3_ composed of flower-like hollow structured composites manufactured by a hydrothermal process^21^. P-type MoO_3_ nanostructures were incorporated into n-type TiO_2_ nanofibers via an electrospinning process^22^, which resulted in higher photocatalytic activities due to the heterojunction effect and band gap alignment. α-MoO_3_ nanorods loaded with α-Fe_2_O_3_ nanoparticles on the surface were synthesized via a two-step hydrothermal method by Xiao et al*.* and used in the photodegradation of tetracycline. This led to enhanced performance because the heterojunction between MoO_3_ and Fe_2_O_3_ allowed efficient electron–hole transport with the main participation of hydroxyl radical and hole^23^. Core–shell α-MoO_3_/α-Fe_2_O_3_ nanostructure composites were fabricated by a hydrothermal method and were shown to be a promising xylene sensor with a three times higher response than α-MoO_3_ at 206 °C^24^ due to quick adsorption and desorption at 1D nanostructures and the lowered effective barrier height of the heterojunction. Fe_2_O_3_·*n*MoO_3_ nanowires with urchin-like and bowknot-like nanostructures, exhibiting 97.8% efficiency for Congo red degradation, were also synthesized^25^. Ternary Fe_2_O_3_/MoO_3_/AgBr nanoparticles were synthesized and uncovered the important role of superoxide radicals and holes in the decomposition of AB92 dye, giving a pseudo-first-order rate constant of 18.23 × 10^−2^ min^−1^ for the degradation reaction^26^.

Based on the importance of 1D structures and the enhancement of photodegradation with heterojunction structures, in this study we developed a two-step microwave-assisted synthetic method to synthesize 1D h-MoO_3_ hexagonal prisms decorated with Fe_2_O_3_ nanoparticles. This simple and rapid method for preparing these materials is an energy saving and environmentally friendly approach. Furthermore, the highly efficient photocatalysts, prepared via a microwave-assisted synthetic method, have the potential to be reused, while retaining stability. The synthesized materials were characterized by X-ray powder diffraction (XRD), scanning electron microscopy (SEM), Raman, and UV–Vis diffuse reflectance spectroscopy (DRS). Finally, photodegradation of MB was evaluated for the synthesized composite Fe_2_O_3_/MoO_3_ structure and the photodegradation mechanism was investigated using various types of sacrificial agents.

## Experimental

### Chemicals

All chemicals used in this study were analytical grade and used without further treatment. Ammonium molybdenum tetrahydrate ((NH_4_)_6_Mo_7_O_24_·4H_2_O, AHM, 99.98%) was purchased from Strem Chemicals, Inc. Nitric acid (HNO_3_, 15.7 M) was obtained from J. T. Baker. Iron(III) nitrate nonahydrate (Fe(NO_3_)_3_·9H_2_O, 98^+^%) and benzoquinone (C_6_H_4_O_2_, BQ, 98^+^%) were purchased from Alfa Aesar. Ethanol (C_2_H_5_OH, 95%), hydrogen peroxide (H_2_O_2_, 35%), and ethylenediaminetetraacetate (C_10_H_16_N_2_Na_2_O_8_, EDTA, 99%) were purchased from Sigma–Aldrich. Methylene blue (C_16_H_18_N_3_SCl·3H_2_O, MB, 95%) was purchased from Acros Organics. Isopropanol (C_3_H_7_OH, IPA, 99.8%) was purchased from Macron Fine Chemical.

### Material preparation

The composite Fe_2_O_3_/MoO_3_ material was synthesized by a two-step procedure beginning with the synthesis of metastable h-MoO_3_ hexagonal prisms using a microwave heating method, followed by a mixing calcination process with different amounts of Fe_2_O_3_ decorated onto the MoO_3_.

### Synthesis of h-MoO_3_ by microwave heating

An aqueous solution of 0.04 M AHM was prepared in deionized water and stirred until all AHM had dissolved. Subsequently, 10 mL of 2.2 M HNO_3_ (aqueous solution) was added to the AHM solution and heated in a Milestone STARTS microwave digestion system at 70 °C for 30 min. After centrifugation at 20,000 RPM, the white powder was washed with deionized water and ethanol several times and dried in an oven at 60 °C overnight. The white hexagonal prism of h-MoO_3_ was obtained and is referred to as h-MO in the rest of the paper.

### Synthesis of Fe_2_O_3_/MoO_3_ by mixing calcination

0.176 g of Fe(NO)_3_·9H_2_O was added to 50 mL of deionized water and stirred until dissolved. This solution is denoted as solution A. Four different volumes (1, 4, 7, and 14 mL) of solution A were then diluted with deionized water to a final volume of 100 mL. 0.15 g of h-MoO_3_ was added to each solution and stirred at 40 °C for 90 min. An orange powder was obtained after centrifuging at 20,000 RPM and was washed with deionized water and ethanol several times and then dried in an oven at 60 °C overnight. Finally, the dried sample was calcinated at 400 °C for 2 h in air, and a brown-yellow Fe_2_O_3_/MoO_3_ composite was obtained. The products were named h-MO-1, h-MO-4, h-MO-7, and h-MO-14, which corresponds to the volume of solution A used during preparation. h-MoO_3_ calcinated at 400 °C for 2 h in air was named h-MO-0. For comparison, a pure α-Fe_2_O_3_ sample was processed using this procedure in the absence of h-MoO_3_.

### Material characterization

The crystal structures and phases of the synthesized products were characterized by a Rigaku Multiflex X-Ray diffractometer using CuKα as the radiation source (λ = 1.5418 Å). 2θ ranged from 5° to 60° with a scan rate of 1°/min. The XRD patterns were compared with the Joint Committee on Powder Diffraction Standards’ (JCPDS) database entries for identification. The morphologies of the synthesized products were recorded using a JOEL JSM-6330TF field emission scanning electron microscope and JEOL JEM-3010 analytical scanning transmission electron microscope. A PTT BWII RAMker micro-Raman spectrometer was used to obtain the Raman spectrum of the synthesized products with a 633 nm He–Ne Laser source, with scanning done from 70 to 1100 cm^−1^. Surface elemental analysis was executed using X-ray photoelectron spectroscopy (XPS) using a ULVAC-PHI PHI 5000 Versaprobe II instrument. The UV–Vis absorbance of MB was monitored at fixed intervals using a Hitachi U-3010 UV–Vis spectrophotometer in the wavelength range of 400–800 nm with a 300 nm/min scan rate. The UV–Vis absorption spectra of the powdered samples were recorded in the range of 200–800 nm with an AvaSpec-ULS2048 UV–Vis spectrometer at room temperature. The photoluminescence (PL) emissions of the powder samples were measured by UniRam. Electrochemical impedance spectra (EIS) were analyzed by CHI Model 704A.

### Photocatalytic degradation

The prepared samples were investigated for photocatalytic degradation of MB in aqueous solution at room temperature using a PR-2000 photochemical reactor under visible-light irradiation. The following experimental procedure was used: 20 mg of catalyst was suspended in 100 mL of 8 ppm MB aqueous solution. 0.1 mL of 35 wt% H_2_O_2_ was added and the sample was stirred in the dark for 60 min to reach sorption–desorption equilibrium. The photocatalytic reaction was then performed by irradiating the mixture with 420 nm light using a 128 W fluorescent tube light. During irradiation, 3 mL of solution was removed at designated time intervals and centrifuged at 20,000 rpm for 5 min. The supernatant solutions were analyzed by a Hitachi U-3010 UV–Vis spectrophotometer, and the spectra were recorded with the characteristic absorption peak of MB at λ = 664 nm.

## Results and discussion

### XRD studies

**Figure 1:**
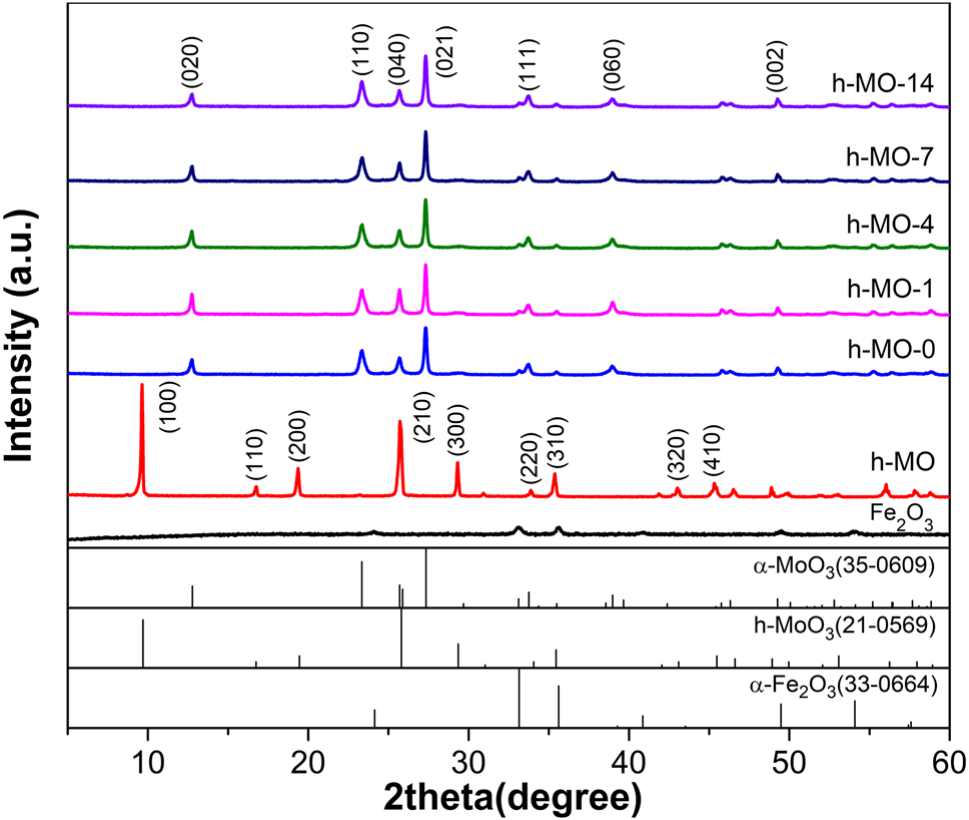
XRD patterns of Fe_2_O_3_–MoO_3_ composites with different Fe_2_O_3_ concentration, and standard XRD patterns of α-MoO_3_ (JCPDS 05-0508), h-MoO_3_ (JCPDS 21-0569) and α-Fe_2_O_3_ (JCPDS 33-0664).

The crystal structures of the different samples were characterized by XRD and are shown in Fig. 1. From the XRD spectrum of pure Fe_2_O_3_ sample, peaks were identified at 2θ = 24.1°, 33.1°, 35.6°, 40.8°, 49.4°, and 54.0°. By comparison with the JCPDS database, we confirmed that rhombohedral α-Fe_2_O_3_ (JCPDS card no. 33-0664) crystals had formed. The XRD diffraction peaks of the h-MO sample were at 2θ = 9.6°, 16.7°, 19.4°, 25.8°, 29.3°, and 35.4°, which were confirmed to be hexagonal MoO_3_ (JCPDS card no. 21-0569) with a (100)-preferred orientation. After mixing of h-MO with various concentrations of Fe(NO)_3_ aqueous solution and calcinating, the crystal structure was altered. The XRD patterns of h-MO-0, h-MO-1, h-MO-4, h-MO-7, and h-MO-14 were identified to be in the orthorhombic α-MoO_3_ crystal phase (JCPDS card no. 35-0609) with a (021)-preferred orientation. However, in the XRD of h-MO-*x* samples, none of the peaks belonging to α-Fe_2_O_3_ could be identified. This could possibly be due to the low amount of decorated Fe_2_O_3_ in the sample or poor crystallinity of Fe_2_O_3_ synthesized through this method^27^.

### SEM analyses

**Figure 2:**
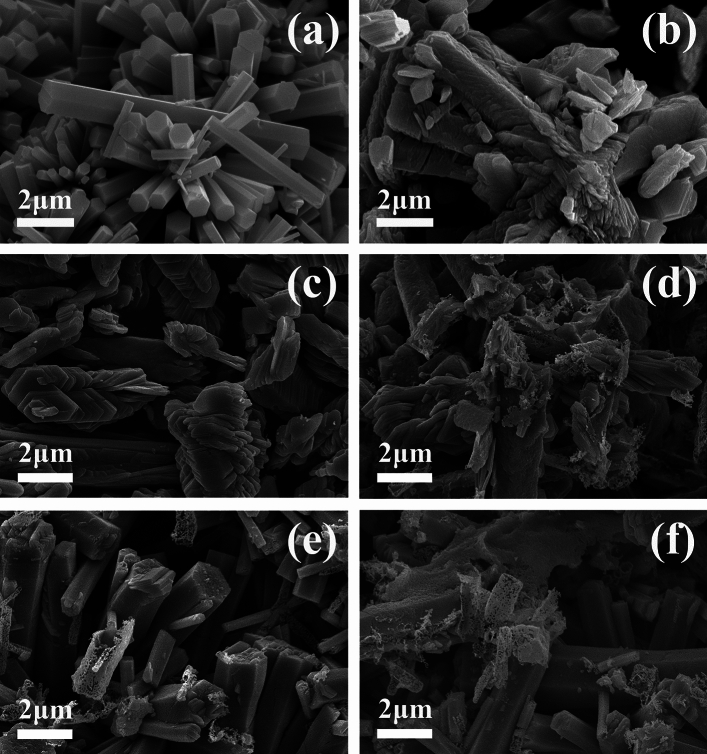
SEM Images of (**a**) h-MO, (**b**) h-MO-0, (**c**) h-MO-1, (**d**) h-MO-4, (**e**) h-MO-7, and (**f**) h-MO-14.

The SEM images of h-MO (a), h-MO-0 (b), and various Fe_2_O_3_/MoO_3_ composites (c to f), are shown in Fig. 2. In Fig. 2a, h-MO takes the form of 1D hexagonal prism rods. The individual rods are polygon-like crystals with hexagonal cross sections and well-developed facets. Impurity particles are not apparent, and the average diameter is about 500–800 nm, with the length approximately between 5 and 10 µm. From Fig. 2b, the h-MoO_3_ sample calcinated at 400 °C for 2 h in air (h-MO-0) was found to transform to the orthorhombic α-MoO_3_ crystal phase, and sheet-like structures are observed instead of hexagonal prism nanorods. As the amount of Fe_2_O_3_ decoration increases (Fig. 2c–f, from h-MO-1 to h-MO-14), the morphology of the Fe_2_O_3_/MoO_3_ composites evolved from sheet- or plate-like to hexagonal prism rods. This indicated that despite the increased amount of Fe_2_O_3_ (*i.e.*, h-MO-7 and h-MO-14) decorated on the surface of the original h-MO hexagonal prism rod, the rod-like morphology is retained even as the crystal phase of MoO_3_ transformed from h-MoO_3_ to α-MoO_3,_ as observed from the XRD data. The phase transformation from h-MoO_3_ to α-MoO_3_ can be explained by a dissolution–recrystallization mechanism^28^, where the metastable h-MoO_3_ rod dissolved partially, breaking the zigzag chains of the cis-interlinked MoO_6_ octahedron, and recrystallizing to form the α-MoO_3_ sheet that is constructed by the corner-sharing distorted MoO_6_ octahedron with van der Waals forces. However, with Fe_2_O_3_, the hexagonal prism shapes are preserved while phase transformation takes place, differing from a previous study^14^ where the h-MoO_3_ rod dissolved and recrystallized, forming α-MoO_3_ nanobelts at 400 °C. As the concentration of Fe_2_O_3_ increased, we observed some nanoparticles sitting on the surface of the MoO_3_ hexagonal prisms.

### TEM analyses

**Figure 3:**
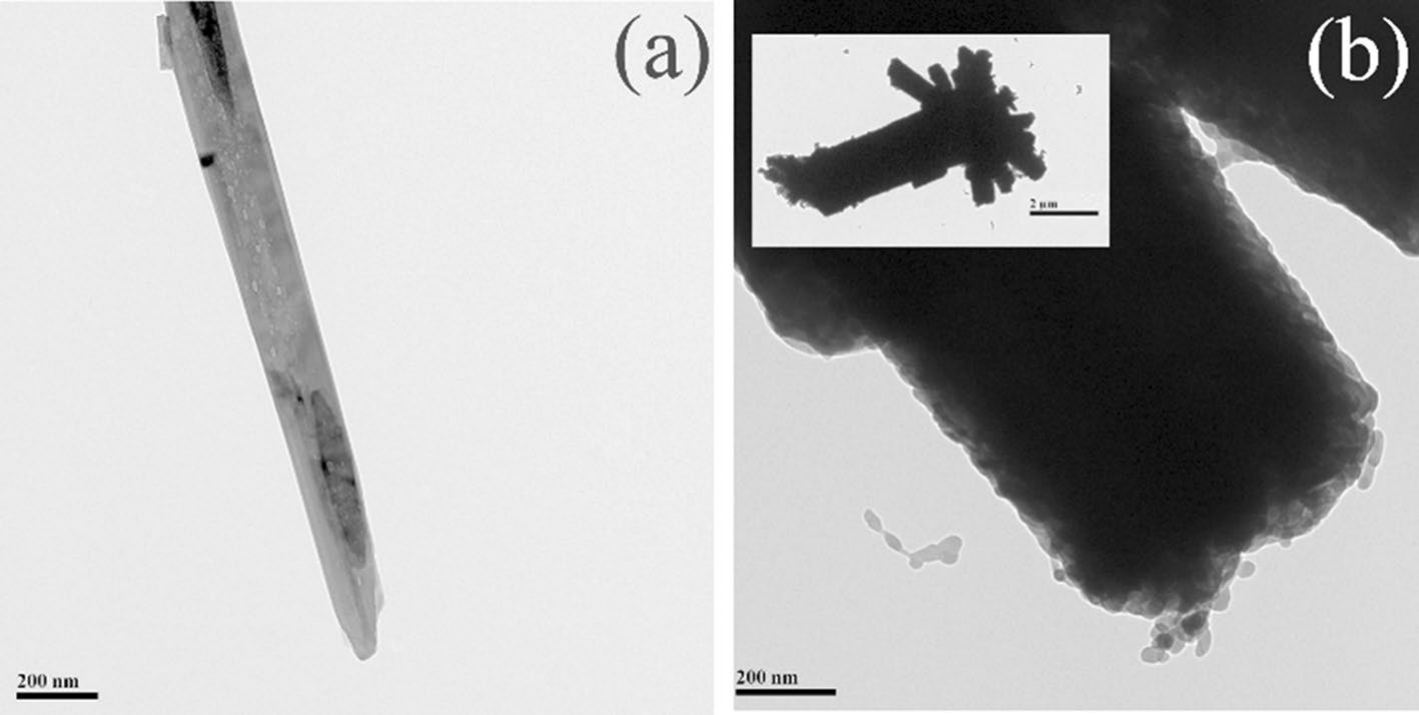
TEM images of (**a**) h-MO-0 and (**b**) h-MO-7.

The morphology of h-MO-0 (a) and h-MO-7 (b) was investigated by TEM and shown in Fig. 3, respectively. All are rod-shaped, but they differ in size. When h-MO is calcinated without Fe_2_O_3_ added to form h-MO-0, the size changes from microrods of approximately 1 μm in length and 200 nm in width (Fig. S1) to nanorods ranging from 500 to 1000 nm in length and 100–200 nm in width. However, the rods become wider with an increasing amount of Fe_2_O_3_. Meanwhile, we observed through EDS (Fig. S1) that only Mo and O are present in h-MO-0, while in h-MO-7, Mo, O, and Fe are present. Moreover, Fe was located in the outer layer. It can be concluded that when a sufficient amount of Fe_2_O_3_ is added, it can completely decorate the surface of MoO_3_ rods. After calcination, although the phase of h-MO-7 has been transferred to α-MoO_3_, the original rod-shaped morphology of h-MO is preserved. These results agree with SEM analysis.

### Raman analyses

**Figure 4:**
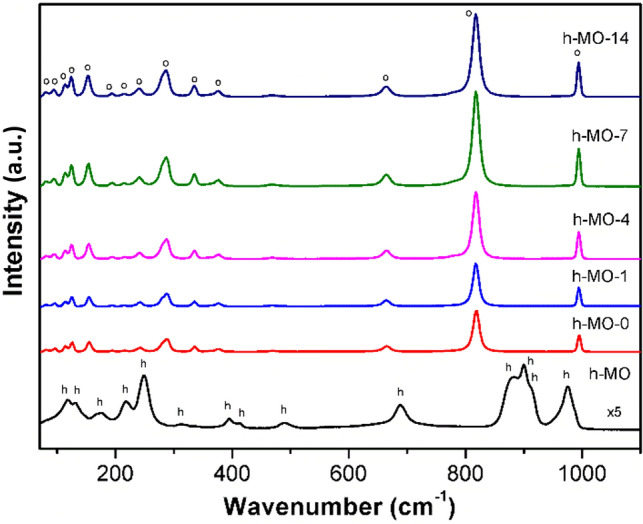
Raman spectra of the different samples.

The Raman spectra of all products are shown in Fig. 4. For h-MO, the observed peaks at 118, 131, 176, 218, and 248 cm^−1^ are all indexed to h-MoO_3_^29^, which corresponds to the skeletal modes of tetrahedral MoO_4_. The peaks at 313, 395, 413, 489, and 689 cm^−1^ correspond to O–Mo–O vibrations, and the peaks at 882, 900, 911, and 974 cm^−1^ correspond to the Mo=O bond. The Raman results agreed with the previous XRD and SEM findings that the h-MO sample is in the h-MoO_3_ hexagonal prism form. For the h-MO-0, h-MO-1, h-MO-4, h-Mo-7, and h-MO-14 samples, the peaks located at 81, 94, 113, 124, 152, and 213 cm^−1^ are assigned to α-MoO_3_, which corresponds to MoO_4_ translational and rotational chain modes. The 195, 241, and 286 cm^−1^ peaks correspond to O=Mo=O twisting and wagging modes. Peaks at 336, 375, 468, and 663 cm^−1^ correspond to O–Mo–O bending, scissoring, stretching and bending, and stretching modes, respectively. Finally, the sharp and intense peaks at 818 and 994 cm^−1^ correspond to Mo=O stretching modes^30^. The Raman spectra results indicate that the MoO_3_ orthorhombic phase formed after the calcination process, even though the heating temperature of 400 °C was below the phase transformation temperature of 419 °C^31^ or 430 °C^32^ for metastable h-MoO_3_ to stable α-MoO_3_.

### XPS analyses

**Figure 5:**
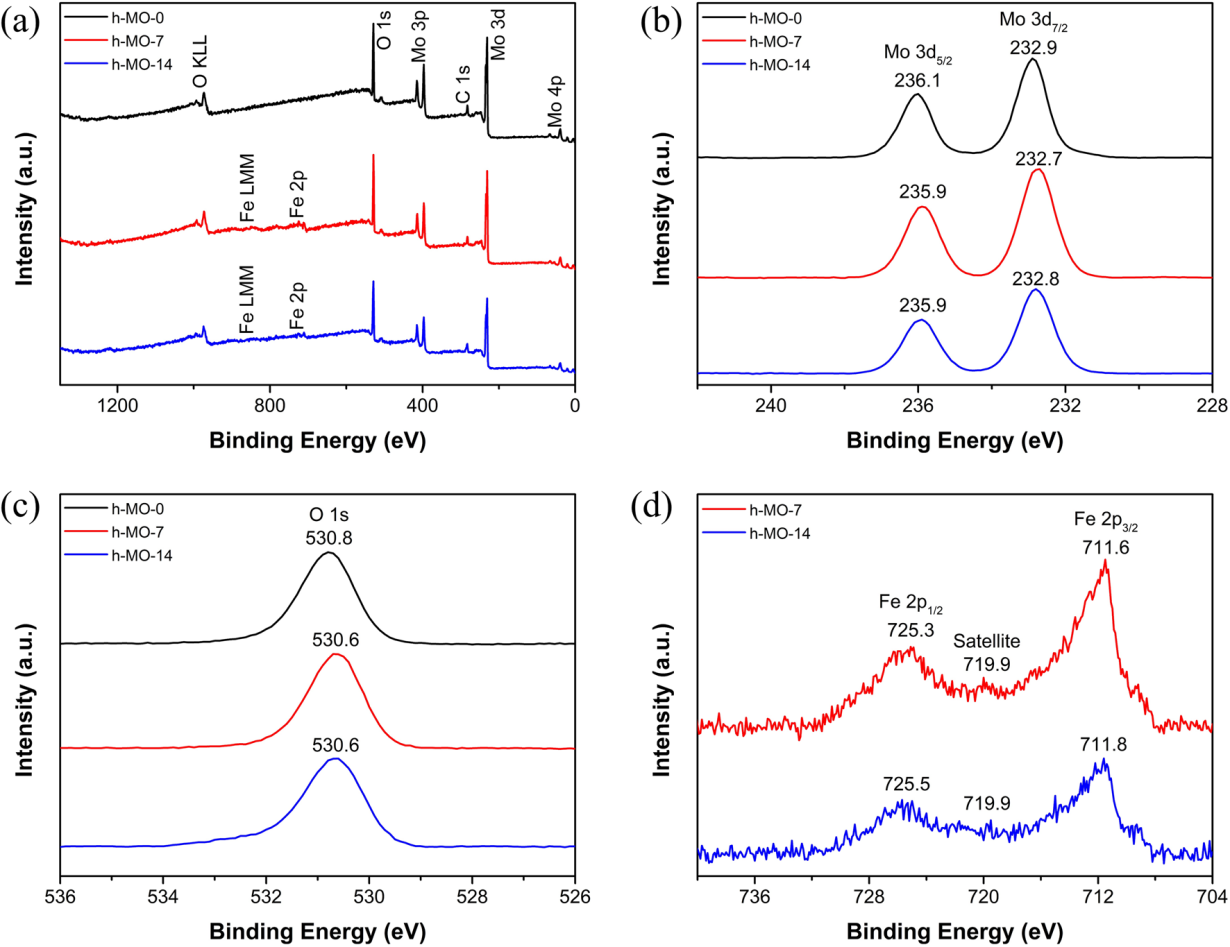
(**a**) XPS survey, (**b**) Mo 3d, (**c**) O 1s, and (**d**) Fe 2p spectra.

XPS spectra were used to analyze the chemical components and valence states of the prepared samples. As shown in Fig. 5a, the elements Mo, O, and C are present in the h-MO-0 sample. However, in addition to the above elements, there is also presence of Fe in h-MO-7 and h-MO-14 samples. Figure 5b reveals the two peaks of Mo 3d located at around 236.1 and 232.9 eV, which correspond to Mo 3d_3/2_ and Mo 3d_5/2_, respectively, suggesting the presence of Mo in the 6 + oxidation state. For the O 1s spectra shown in Fig. 5c, the peak observed at around 530.8 eV can be assigned to oxygen present in the lattice. From the results above, we can confirm that the samples all contain MoO_3_. In Fig. 5d, the Fe 2p spectra consist of two peaks at around 725.3 and 711.6 eV corresponding to Fe 2p_1/2_ and Fe 2p_3/2_, respectively. Furthermore, a satellite peak at 719.9 eV characteristic of Fe^3+^ is revealed; this indicates that the h-MO-7 and h-MO-14 samples also contain Fe_2_O_3_. Moreover, the chemical shift in Fig. 5b,c can be attributed to the interaction between MoO_3_ and Fe_2_O_3_, where new bonds were formed to form the heterojunction, which enhances photocatalytic efficiency^33^.

### Optical properties

**Figure 6:**
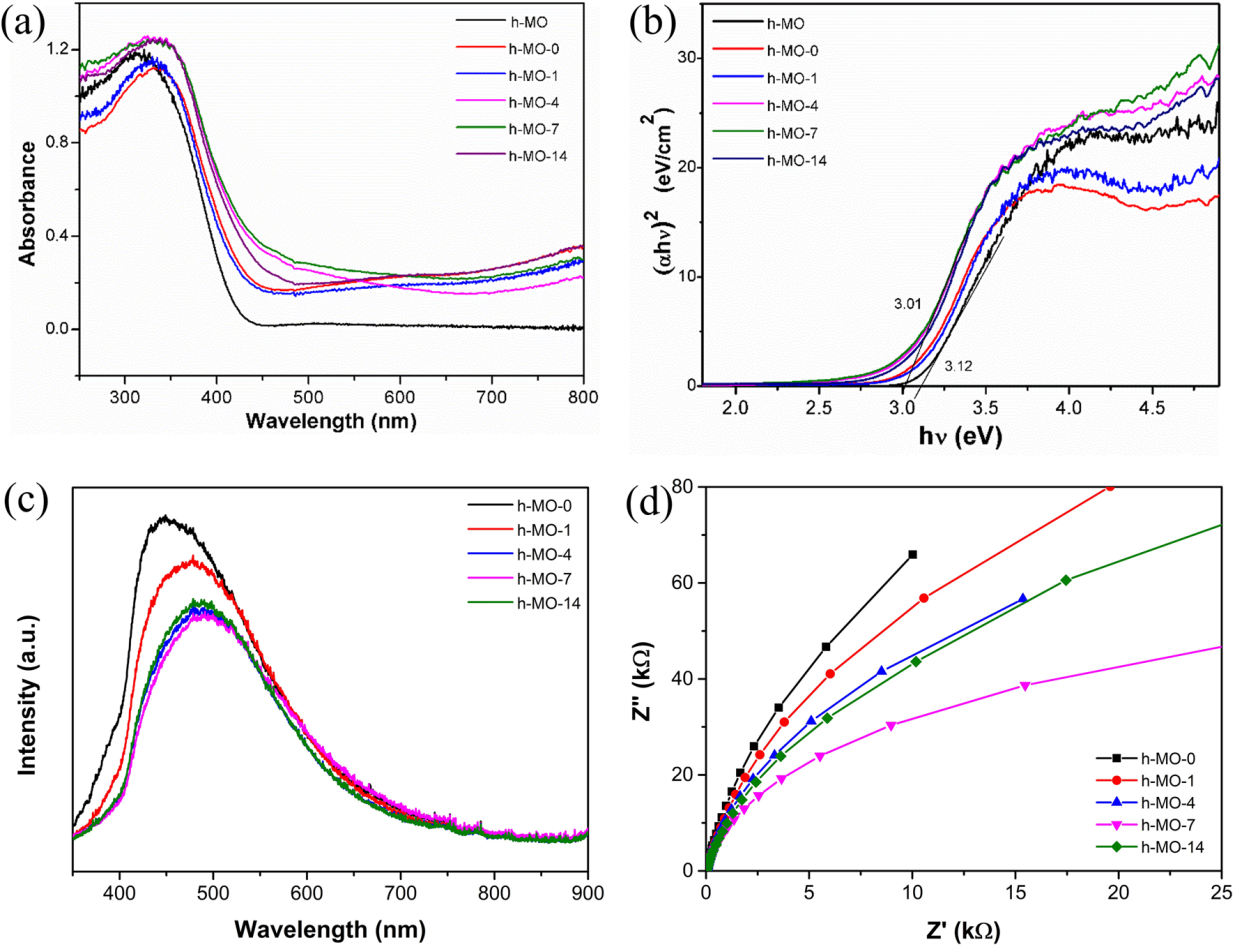
(**a**) UV–Vis spectra, (**b**) Tauc plot of different samples, (**c**) PL spectra, and (**d**) EIS plots.

The photoabsorption properties of the samples were characterized by UV–Vis DRS. From Fig. 6a, the absorption edge of h-MO is around 440 nm, and the absorption maximum is around 310 nm. For all h-MO-*x* samples, we observed red-shifted spectra compared with the h-MO sample. We also found that both the position of the absorption peak and absorption onset are red-shifted as the amount of Fe_2_O_3_ increased. However, h-MO-14 gave a similar result to h-MO-4, which was an exception. The band gap energy can be determined on the basis of the Tauc plot method^34–36^:

1 $$\left( {\alpha hv} \right) = A_{n} \left( {hv - E_{g} } \right)^{n}$$

where α, *h*, ν, *E*_*g*_, and *A*_*n*_ are the absorption coefficient, Planck constant, light frequency, optical energy gap, and the probability parameter for the transition, respectively. In addition, *n* is determined by the type of optical transition in a semiconductor, where *n* = 1/2 for allowed direct transitions and *n* = 2 for indirect transitions^37^. For MoO_3_, a direct transition gap is observed, and so the value of n is set to 2.

2 $$\left( {\alpha hv} \right)^{2} = A\left( {hv - E_{g} } \right)$$

The gap energy can be given from the linear region of the plot (αhν)^2^ versus hν. From Fig. 6b, the band gap energies of h-MO, h-MO-0, h-MO-1, h-MO-4, h-MO-7, and h-MO-14 were estimated to be 3.12, 3.06, 3.08, 3.03, 3.01, and 3.03 eV, respectively. With increasing Fe_2_O_3_ content, the gap energy lowers from 3.06 to 3.01 eV, except for the gap energy of h-MO-14 (3.03 eV), which is the same as h-MO-4. The smaller gap energy enhances visible-light absorption and leads to more photogenerated electron–hole pairs. Therefore, the h-MO-*x* composites have improved photocatalytic performance compared to the hexagonal MoO_3_.

PL was used to measure the recombination rate of photogenerated charge carriers in the metal oxide semiconductors. In Fig. 6c, h-MO-0 displayed the strongest emission intensity. As the Fe_2_O_3_ content increased, the emission intensities are lower than h-MO-0 (pure MoO_3_). h-MO-0 and h-MO-7 demonstrated the lowest peak, indicating that the separation efficiency of photogenerated charge carriers in the composites significantly increased due to the formation of the heterojunction, resulting in improved photocatalytic performance. However, the emission intensity of h-MO-14 was higher than that of h-MO-7 since an excess of Fe_2_O_3_ covering the active sites of MoO_3_ prevented MoO_3_ from forming photogenerated carriers. Therefore, the separation efficiency is decreased compared to h-MO-7^19,38,39^.

The charge transfer ability of the synthesized samples was measured using EIS, as shown in Fig. 6d. The arc radius of h-MO-7 was the smallest among the samples tested. A smaller arc radius indicates a more efficient charge transfer, suggesting that h-MO-7 facilitates faster electron transfer across the heterojunction compared to the other samples. This result is consistent with the PL results, indicating that when the material possesses better charge transfer between heterojunctions there is an increase in the electron–hole separation efficiency. This characteristic is beneficial for enhancing photocatalytic efficiency^40–43^.

### Evaluating the photocatalytic degradation of MB

**Figure 7:**
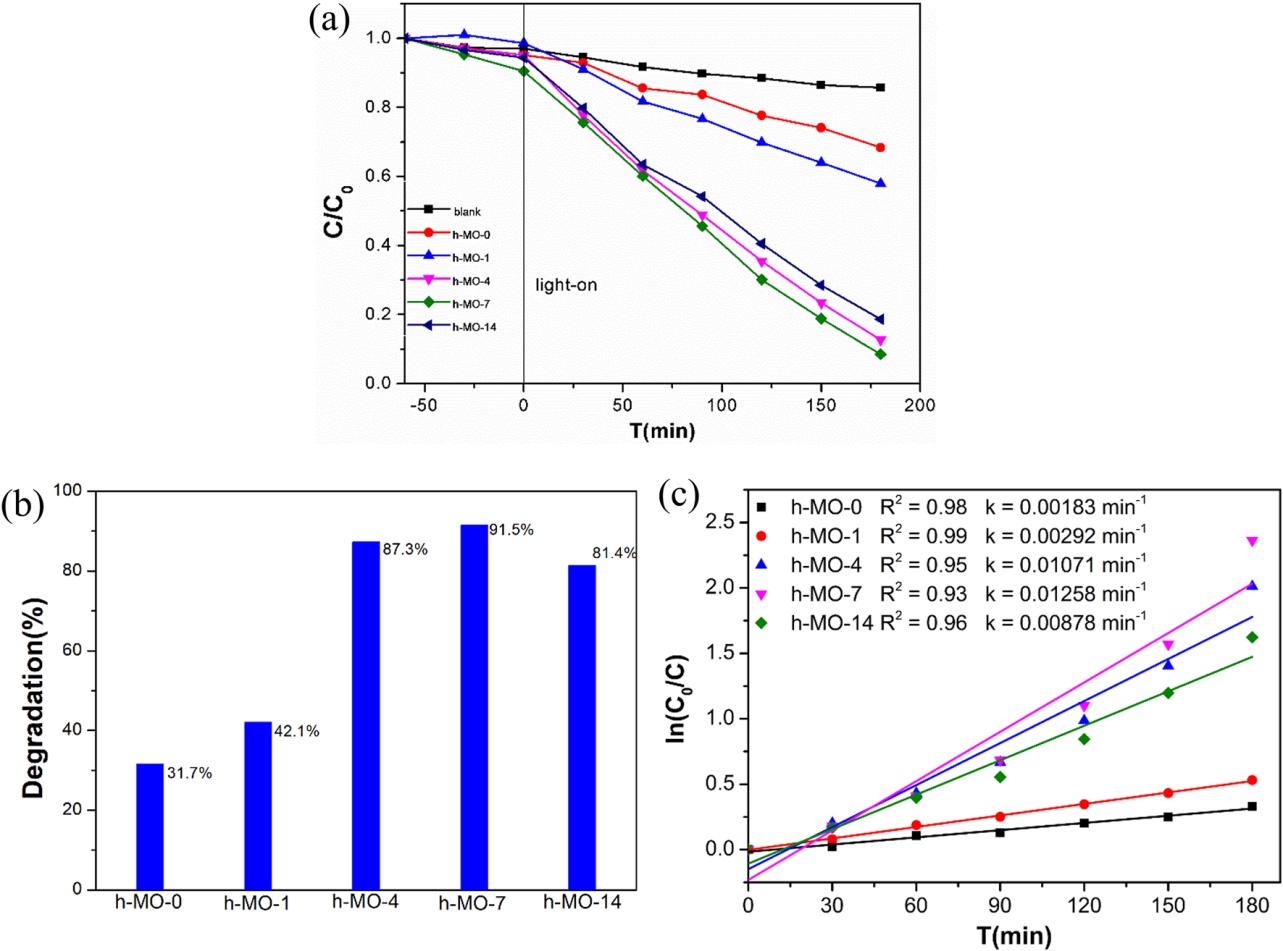
(**a**) Photocatalytic degradation, (**b**) percentage of photodegradation, and (**c**) rate kinetics of Methylene Blue degradation under 420 nm irradiation.

The photocatalytic performance of the synthesized samples was investigated with 420 nm visible light, as shown in Fig. 7a. As a baseline, a dark adsorption experiment was performed by stirring for 60 min to achieve a pre-equilibrium state before visible-light irradiation. After 180 min of irradiation, we found that pure α-MoO_3_ (h-MO-0) only degraded 31.7% of MB, while the Fe_2_O_3_/MoO_3_ composites, h-MO-1, h-MO-4, h-MO-7, and h-MO-14, degraded 42.1, 87.3, 91.5, and 81.4% of MB, respectively (Fig. 7b). The degree of MB degradation increased as the amount of Fe_2_O_3_ increased, except for the case of h-MO-14. h-MO-14 had higher Fe_2_O_3_ content but lower efficiency than h-MO-7 probably because Fe_2_O_3_ nanoparticles are distributed and coated on the surface of the MoO_3_ rods, where electrons recombine with holes on the surface of Fe_2_O_3_, making reactions with hydrogen peroxide difficult^22,44^. h-MO-7 has the greatest degradation efficiency of 91.5% which is about three times larger than that of undecorated MoO_3_ (h-MO-0), which could be explained by the lower gap energy, as indicated by the UV–Vis analysis.

To further investigate the photocatalytic activity of the prepared samples, the Langmuir–Hinshelwood kinetics model was applied to evaluate the degradation rate and expressed in the following equation ^45–47^:

3 $$\ln \frac{{C_{0} }}{C} = kt$$

where *C*_*o*_ is the initial concentration of MB, *C* is the concentration of MB at time *t* (in minutes), and *k* is the unimolecular photodegradation rate constant. Photodegradation rate constants were calculated by plotting ln(*C*_0_/*C*) versus irradiation time and given in Fig. 7c. We found that the rate constant of h-MO-7 (0.01288 min^−1^) is about seven times that of the undecorated h-MO-0 sample (0.00183 min^−1^). Compared with other research (Table S1), these catalysts show good performance^14,23,31,48,49^.

### Fenton-like processes

**Figure 8:**
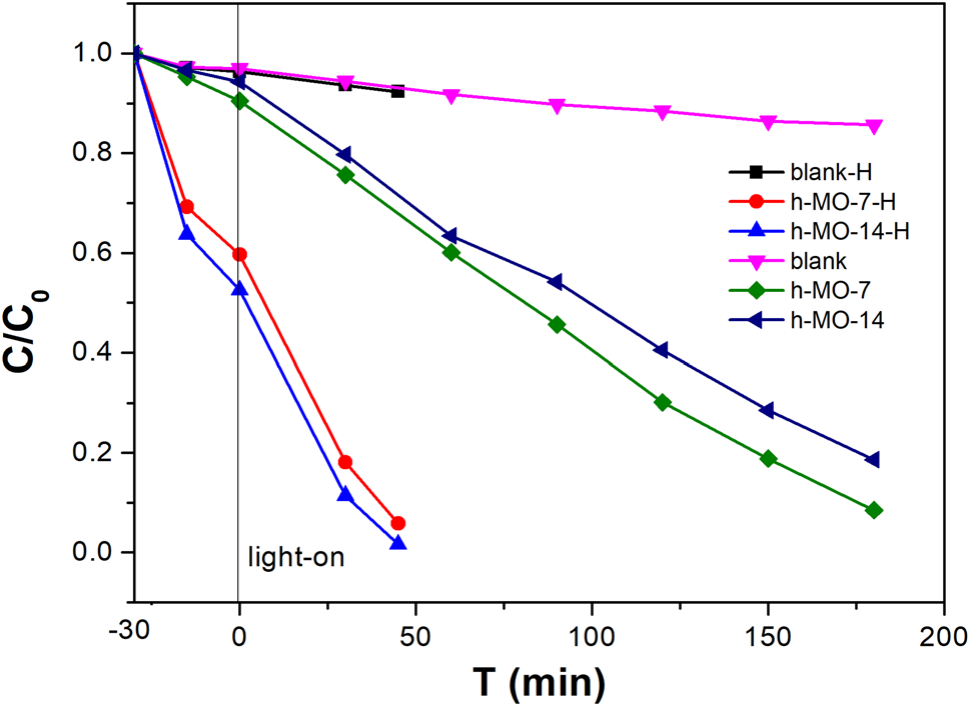
Photocatalytic degradation of methylene blue with addition of H_2_O_2_ under 420 nm irradiation.

From Fig. 8, it can be seen that addition of 5 mL of hydrogen peroxide to the h-MO-14 (denoted as h-MO-14-H) and h-MO-7 (denoted as h-MO-7-H) samples further enhanced the photodegradation process. Without additional H_2_O_2_, MB is fully degraded after 150 min with h-MO-7 and h-MO-14. However, for the h-MO-7-H and h-MO-14-H samples, MB is fully degraded in about 50 min. This might be due to both the Fenton reaction,

$${\text{Fe}}^{{{2} + }} {\text{ + H}}_{{2}} {\text{O}}_{{2}} \to {\text{Fe}}^{{{3} + }} + \cdot {\text{OH }} + {\text{OH}}^{ - }$$

and the Fenton-like mechanism taking place:

$${\text{Fe}}^{{{3} + }} + {\text{ H}}_{{2}} {\text{O}}_{{2}} \to {\text{ FeOOH}}^{{{2} + }} + {\text{ H}}^{ + }$$

$${\text{FeOOH}}^{{{2} + }} \to {\text{ Fe}}^{{{2} + }} + \, \cdot {\text{OH}}$$

$${\text{Fe}}^{{{3} + }} + \cdot {\text{OOH}} \to {\text{Fe}}^{{{2} + }} + {\text{ O}}_{{2}} + {\text{ H}}^{ + }$$

where more hydroxyl radicals are produced to fragmentize the MB^50–52^ due to the higher content of Fe_2_O_3_ in h-MO-14-H.

### Effect of sacrificial agents

**Figure 9:**
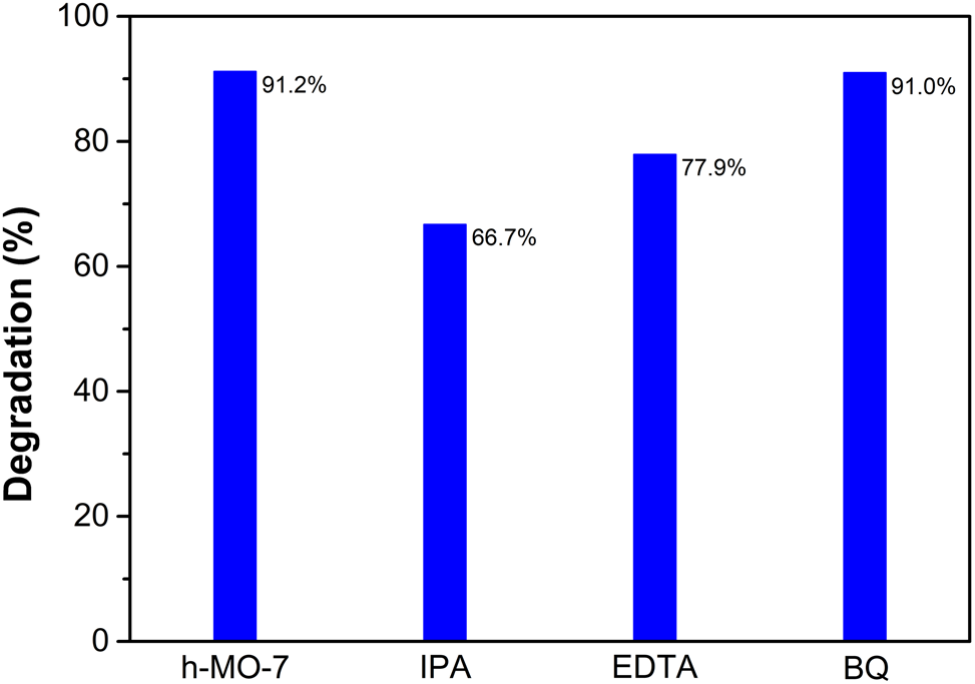
Photodegradation percentage with different sacrificial agents.

There are four possible factors involved in the photodegradation of MB: electrons, holes, hydroxyl radicals (·OH), and superoxide ions (·O_2_^−^). Sacrificial agents were added to identify factors in the degradation reaction. In our kinetic experiments, H_2_O_2_ served as an electron sacrificial agent that consumes electrons, and so no further investigation was conducted on the effect of electrons. IPA served as the sacrificial agent for hydroxyl radicals, while EDTA and BQ were the sacrificial agents for holes and superoxide ions, respectively^53,54^. In Fig. 9, when adding IPA to the h-MO-7 sample containing 0.1 mL H_2_O_2_, the efficiency decreased significantly due to the consumption of hydroxyl radicals. Adding EDTA, which consumes holes, also decreased the efficiency of photodegradation. The efficiency decreased only by a small amount with the addition of BQ. From these tests, we conclude that production of ·OH radicals is the primary factor in the photodegradation mechanism and that hole generation is a secondary factor.

### Photodegradation mechanism

**Figure 10:**
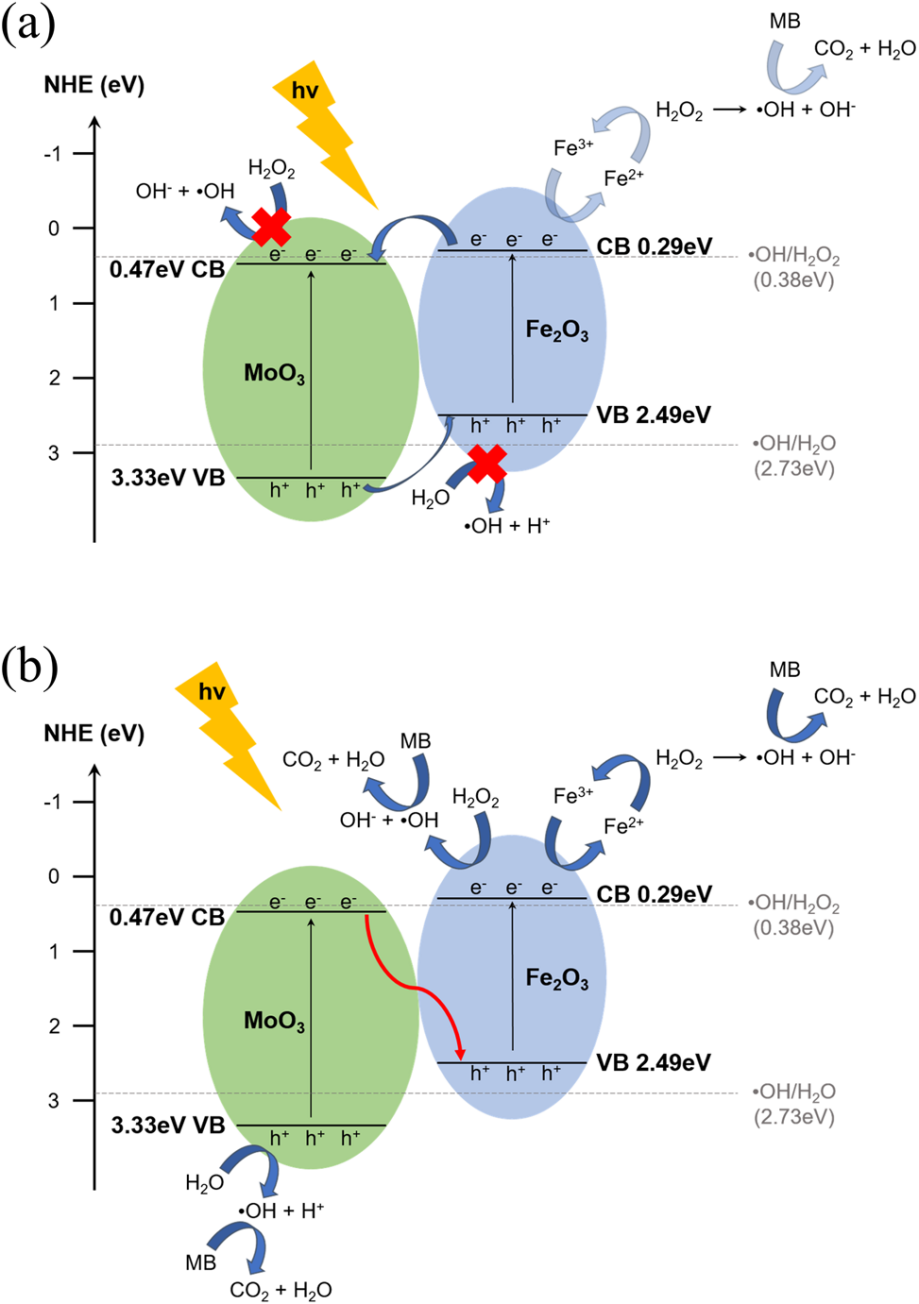
(**a**) Type-II and (**b**) Z-Scheme diagram of proposed photocatalytic mechanism and energy band gap of MoO_3_ and Fe_2_O_3_.

The enhanced photocatalytic activity of the Fe_2_O_3_/MoO_3_ composites can be explained by the relative band positions of the Fe_2_O_3_/MoO_3_ composites. The band energies of two metal oxide semiconductors can be estimated by the following expressions^55^:

4 $$E_{{{\text{VB}}}} { = }X - E_{0} + 0.5E_{g}$$

5 $$E_{{{\text{CB}}}} { = }E_{VB} - 0.5E_{g}$$

where *E*_VB_ is the valence band edge potential, *Χ* is the geometric mean of the constituent atoms’ electronegativities, *E*_0_ is the energy of the free electrons on the hydrogen scale (~ 4.50 eV), and *E*_g_ is the band gap of the semiconductor. On the basis of previous reports, *E*_VB_ and *E*_CB_ of Fe_2_O_3_ were calculated to be 2.49 and 0.29 eV, respectively, and the *E*_VB_ and *E*_CB_ values of MoO_3_ were found to be 3.33 and 0.47 eV^33,53^, respectively. As shown in Fig. 10a,b, type II and Z-scheme modes are two possible synergistic modes in this photocatalytic process. From Fig. 10a, it can be deduced that e^**−**^ on the CB of Fe_2_O_3_ would flow to the CB of MoO_3_, and h^+^ on the VB of MoO_3_ would transfer to the VB of Fe_2_O_3_. However, the more negative potential of **·**OH/H_2_O_2_ (0.38 eV vs. NHE) than the *E*_CB_ of MoO_3_ and the more positive potential of **·**OH/H_2_O (2.73 eV vs. NHE) than the *E*_VB_ of Fe_2_O_3_ make formation of hydroxyl radicals difficult. Moreover, the removal of e^−^ on the CB of Fe_2_O_3_ also interferes with the Fenton-like reaction, contradicting our earlier results that indicate that the photodegradation reaction involves a Fenton-like mechanism when H_2_O_2_ is added. Thus, a type-II-mode-based combination between MoO_3_ and Fe_2_O_3_ is excluded. In a Z-scheme mode, e^**−**^ on the CB of MoO_3_ can combine with h^+^ on the VB of Fe_2_O_3_ directly and prevent the remaining e^**−**^ and h^+^ from recombining. Because the E_CB_ of Fe_2_O_3_ is more negative than the potential of ·OH/H_2_O_2_ (0.38 eV vs. NHE) and the E_VB_ is more positive than the potential of ·OH/H_2_O (2.73 eV vs. NHE), the photodegradation reactions can be carried out. Furthermore, preservation of e^**−**^ on the CB of Fe_2_O_3_ improves the probability of the Fenton-like mechanism, consistent with previous results. Therefore, we conclude that the Z-scheme mode is the mechanism that takes place^56–59^.

### Catalytic recycle

**Figure 11:**
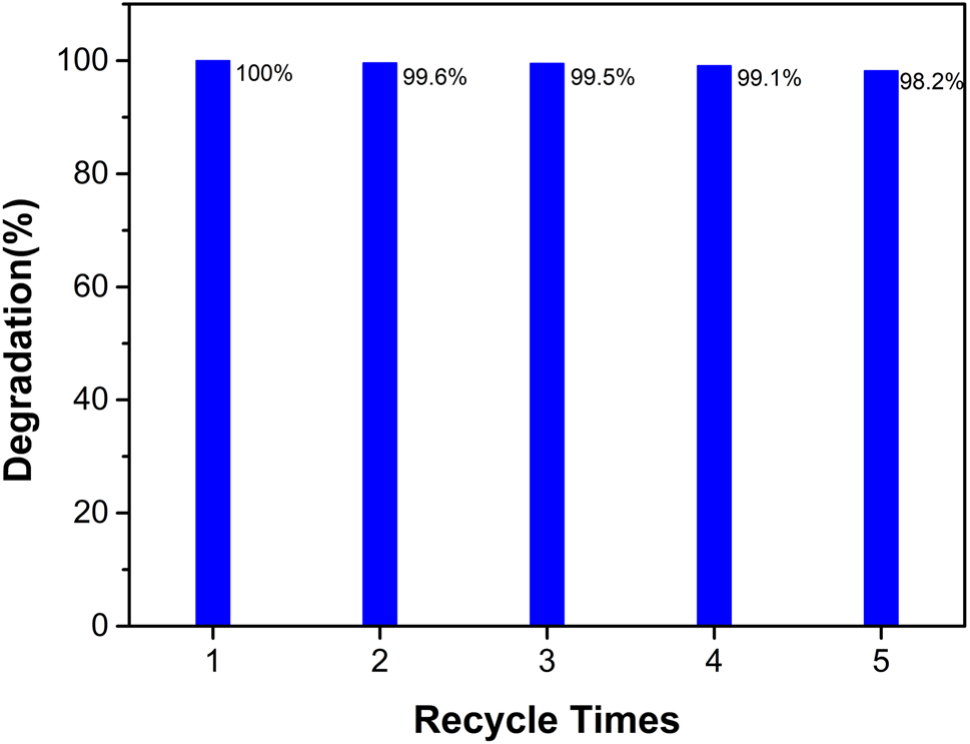
Efficiency of photodegradation of methylene blue for the five recycling runs.

The stability of the h-MO-7 catalyst was investigated by a recycle test. We added 30 mg of sample into 100 mL of 8 ppm MB aqueous solution with 0.1 mL of 35 wt % hydrogen peroxide. The mixture was stirred in the dark for 60 min to achieve adsorption–desorption equilibrium. The mixture was irradiated with 128 W, 420 nm fluorescent light for 180 min. The sample was dried in an oven and reused for the same reaction five times. As shown in Fig. 11, we found that the efficiency of photodegradation was about 98% after the fifth cycle, which could be due to sample loss during the recycling process. Thus, the stability and potential for reusing the h-MO-7 and Fe_2_O_3_/MoO_3_ composite catalysts are promising.

## Conclusion

We have successfully synthesized an Fe_2_O_3_/MoO_3_ composite semiconductor photocatalysts using a two-step method that consists of microwave synthesis and a mixing calcination process. With a sufficient amount of Fe_2_O_3_, the shape of the Fe_2_O_3_-decorated hexagonal rod prism structure was preserved even though the crystal phase transformed from hexagonal h-MoO_3_ to orthorhombic α-MoO_3_. The synthesized composites were applied in the photodegradation of MB, and the best efficiency of 91.5% was found to belong to h-MO-7, compared to 31.7% found for hexagonal MoO_3_. This might be due to reduction of the gap energy, in addition to the production of hydroxyl radicals. OH radical production is a key factor in the photodegradation reaction, as confirmed by adding IPA, EDTA, and BQ sacrificial agents. Furthermore, photodegradation efficiency could be further improved by a Fenton-like process, which was identified by adding H_2_O_2_. Finally, there is a possibility of reusing the Fe_2_O_3_/MoO_3_ rod composites because they remain highly efficient after recycling. These results imply that Fe_2_O_3_/MoO_3_ rod composites (h-MO-7) are prospective materials for removing pollutants from industrial wastewater.

### Supplementary Information

Supplementary Information.

## Data Availability

All data analyzed during the study are included in this published article.

## References

[1] Chong MN, Jin B, Chow CWK, Saint C (2010). Recent developments in photocatalytic water treatment technology: A review. Water Res..

[2] Zhou H, Qu Y, Zeid T, Duan X (2012). Towards highly efficient photocatalysts using semiconductor nanoarchitectures. Energy Environ. Sci..

[3] Hoffmann MR, Martin ST, Choi W, Bahnemannt DWA (1995). Environmental applications of semiconductor photocatalysis. Chem. Rev..

[4] Govindaraj T, Mahendran C, Manikandan VS, Suresh R (2020). One-pot synthesis of tungsten oxide nanostructured for enhanced photocatalytic organic dye degradation. J. Mater. Sci. Mater. Electron..

[5] Wu Z (2009). The fabrication and characterization of novel carbon doped TiO_2_ nanotubes, nanowires and nanorods with high visible light photocatalytic activity. Nanotechnology.

[6] Kusior A, Michalec K, Jelen P, Radecka M (2019). Shaped Fe_2_O_3_ nanoparticles – synthesis and enhanced photocatalytic degradation towards RhB. Appl. Surf. Sci..

[7] Rahmat M (2019). Highly efficient removal of crystal violet dye from water by MnO_2_ based nanofibrous mesh/photocatalytic process. J. Mater. Res. Technol..

[8] Iqbal M (2017). Influence of Mn-doping on the photocatalytic and solar cell efficiency of CuO nanowires. Inorg. Chem. Commun..

[9] Parthibavarman M, Karthik M, Prabhakaran S (2018). Facile and one step synthesis of WO_3_ nanorods and nanosheets as an efficient photocatalyst and humidity sensing material. Vacuum.

[10] Ejeromedoghene O (2021). Quaternary type IV deep eutectic solvent-based tungsten oxide/niobium oxide photochromic and reverse fading composite complex. New J. Chem..

[11] Lunk H-J (2010). “Hexagonal molybdenum trioxide”—known for 100 years and still a fount of new discoveries. Inorg. Chem..

[12] Manivel A (2015). Synthesis of MoO_3_ nanoparticles for azo dye degradation by catalytic ozonation. Mater. Res. Bull..

[13] Chithambararaj A, Sanjini NS, Velmathi S, Bose AC (2013). Preparation of h-MoO_3_ and α-MoO_3_ nanocrystals: Comparative study on photocatalytic degradation of methylene blue under visible light irradiation. Phys. Chem. Chem. Phys..

[14] Li Z, Ma J, Zhang B, Song C, Wang D (2017). Crystal phase- and morphology-controlled synthesis of MoO_3_ materials. CrystEngComm..

[15] Liu Y, Feng P, Wang Z, Jiao X, Akhtar F (2017). Novel fabrication and enhanced photocatalytic MB degradation of hierarchical porous monoliths of MoO_3_ nanoplates. Sci. Rep..

[16] Wu Z, Dong F, Liu Y, Wang H (2009). Enhancement of the visible light photocatalytic performance of C-doped TiO_2_ by loading with V_2_O_5_. Catal. Commun..

[17] Xu Q, Zhang L, Cheng B, Fan J, Yu J (2020). S-scheme heterojunction photocatalyst. Chem..

[18] Wang Z (2020). Step-scheme CdS/TiO_2_ nanocomposite hollow microsphere with enhanced photocatalytic CO_2_ reduction activity. J. Mater. Sci. Technol..

[19] Wang J, Wang G, Cheng B, Yu J, Fan J (2021). Sulfur-doped g-C_3_N_4_/TiO_2_ S-scheme heterojunction photocatalyst for Congo Red photodegradation. Chin. J. Catal..

[20] Kılıç B, Gedick N, Mucur SP, Hergul AS, Gür E (2015). Band gap engineering and modifying surface of TiO_2_ nanostructures by Fe_2_O_3_ for enhanced-performance of dye sensitized solar cell. Mater. Sci. Semicond. Process..

[21] Wang C (2014). Hierarchical α-Fe_2_O_3_/NiO composites with a hollow structure for a gas sensor. ACS Appl. Mater. Interfaces.

[22] Lu M (2014). p-MoO_3_ nanostructures/n-TiO_2_ nanofiber heterojunctions: Controlled fabrication and enhanced photocatalytic properties. ACS Appl. Mater. Interfaces.

[23] Xiao J, Lv J, Lu Q (2022). Building Fe_2_O_3_/MoO_3_ nanorod heterojunction enable better tetracycline photocatalysis. Metar. Lett..

[24] Jiang D (2015). Xylene gas sensor based on α-MoO_3_/α-Fe_2_O_3_ heterostructure with high response and low operating temperature. RSC Adv..

[25] Su Y (2018). High-concentration organic dye removal using Fe_2_O_3_·3.9MoO_3_ nanowires as Fenton-like catalysts. Environ. Sci. Nano.

[26] Salari H (2020). Fabrication of novel Fe_2_O_3_/MoO_3_/AgBr nanocomposites with enchanced photocatalytic activity under visible light irradiation for organic pollutant degradation. Adv. Powder Tech..

[27] Bai S (2014). Surface decoration of WO_3_ architectures with Fe_2_O_3_ nanoparticles for visible-light-driven photocatalysis. CrystEngComm..

[28] Cheng L, Shao M, Wang X, Hu H (2009). Single-crystalline molybdenum trioxide nanoribbons: Photocatalytic, photoconductive, and electrochemical properties. Chem. Eur. J..

[29] Atuchin VV, Gavrilova TA, Kostrovsky VG, Pokrovsky LD, Troitskaia IB (2008). Morphology and structure of hexagonal MoO_3_ nanorods. Inorg. Mater..

[30] Dieterle M, Weinberg G, Mestl G (2002). Raman spectroscopy of molybdenum oxides Part I. Structural characterization of oxygen defects in MoO_3−x_ by DR UV/Vis, Raman spectroscopy and x-ray diffraction. Phys. Chem. Chem. Phys..

[31] Hu H, Deng C, Xu J, Zhang K, Sun M (2015). Metastable h-MoO_3_ and stable α-MoO_3_ microstructures: Controllable synthesis, growth mechanism and their enhanced photocatalytic activity. J. Exp. Nanosci..

[32] Chithambararaj A, Bose AC (2011). Investigation on structural, thermal, optical and sensing properties of meta-stable hexagonal MoO_3_ nanocrystals of one dimensional structure. Beilstein J. Nanotechnol..

[33] Liu H, Lv T, Zhu C, Zhu Z (2016). Direct bandgap narrowing of TiO_2_/MoO_3_ heterostructure composites for enhanced solar-driven photocatalytic activity. Sol. Energy Mater Sol. Cells.

[34] Wang R, Shi K, Huang D, Zhang J, An S (2019). Synthesis and degradation kinetics of TiO_2_/GO composites with highly efficient activity for adsorption and photocatalytic degradation of MB. Sci. Rep..

[35] Rong J (2016). Preparation of hierarchical micro/nanostructured Bi_2_S_3_-WO_3_ composites for enhanced photocatalytic performance. J. Alloys Compd..

[36] Pandey A, Gupta A, Alam U, Verma N (2024). Construction of a stable S-scheme NiSnO_3_/g-C_3_N_4_ heterojunction on activated carbon fibre for the degradation of glyphosate in water under floe condition. Chemosphere.

[37] Bouzidi A (2003). Effect of substrate temperature on the structural and optical properties of MoO_3_ thin films prepared by spray pyrolysis technique. Mater. Sci. Eng. B.

[38] Pandey A, Alam U, Gupta A, Shim J-J, Verma N (2024). S-scheme heterojunction-mediated hydrogen production over the graphitic carbon nitride-anchored nickel stannate perovskite. Fuel.

[39] Gupta A, Alam U, Verma N (2024). Efficient spatial charge separation in the Ruddlesden-Popper phase-based S-scheme Fe_2_SnO_4_-g-C_3_N_4_ heterojunction for visible light-induced H_2_ generation. Int. J. Hydrogen Energ..

[40] Sin J-C (2024). Design and synthesis of Fe_2_WO_6_/Eu-doped BiOBr nanocomposite: A novel 0D/2D Z-scheme heterojunction system for simultaneous boosted visible-light driven photocatalytic bisphenol A degradation and Cr(VI) reduction. Ceram. Int..

[41] Tan J-H (2023). Fabrication of novel Z-scheme BaFe_2_O_4_/BiOCl nanocomposite with promoted visible light photocatalytic palm oil mill effluent treatment and pathogens destruction. Inorg. Chem. Commun..

[42] Lam S-M (2023). Eminent destruction of organics and pathogens concomitant with power generation in a visible light-responsive photocatalytic fuel cell with NiFe_2_O_4_/ZnO pine tree-like photoanode and CuO/Cu_2_O nanorod cathode. Chemosphere.

[43] Zhao L (2022). Fe_2_WO_6_ coupling on cube-like SrTiO_3_ as a highly active S-scheme heterojunction composite for visible light photocatalysis and antibacterial applications. Environ. Technol. Innov..

[44] Khasawneh OFS, Palaniandy P (2021). Removal of organic pollutants from water by Fe_2_O_3_/TiO_2_ based photocatalytic degradation: A review. Environ. Technol. Innov..

[45] Iqbal M (2019). Photocatalytic degradation of organic pollutant with nanosized cadmium sulfide. Mater. Sci. Energy Technol..

[46] He Y (2014). Enhanced photodegradation activity of methyl orange over Z-scheme type MoO_3_–g-C_3_N_4_ composite under visible light irradiation. RSC Adv..

[47] Dohčević-Mitrović Z (2016). WO_3_/TiO_2_ composite coatings: Structural, optical and photocatalytic properties. Mater. Res. Bull..

[48] Wu R, Song H, Luo N, Ji G (2018). Hydrothermal preparation of 3D flower-like BiPO_4_/Bi_2_WO_6_ microsphere with enhanced visible-light photocatalytic activity. J. Colloid. Interf. Sci..

[49] Zhao H-J (2023). In situ construction of Z-type defective Al_2_O_3_/BiPO_4_ for efficient photocatalytic degradation of organic dyes. Mater. Sci. Eng. B..

[50] Lee H (2013). Kinetic enhancement in photocatalytic oxidation of organic compounds by WO_3_ in the presence of Fenton-like reagent. Appl. Catal. B.

[51] Feng J, Hu X, Yue PL (2004). Novel bentonite clay-based Fe-nanocomposite as a heterogeneous catalyst for photo-Fenton discoloration and mineralization of orange II. Environ. Sci. Technol..

[52] Guo L (2020). Highly efficient visible-light-driven photo-Fenton catalytic performance over FeOOH/Bi_2_WO_6_ composite for organic pollutant degradation. J. Alloys Compd..

[53] Song J, Shi Y, Ren M, Hu G (2014). Synthesis, characterization and excellent photocatalytic activity of Ag/AgBr/MoO_3_ composite photocatalyst. Appl. Phys. A.

[54] Xue Q (2016). Photocatalytic degradation of geosmin by Pd nanoparticle modified WO_3_ catalyst under simulated solar light. Chem. Eng. J..

[55] Zhang J, Zhang L, Shen X, Xu P, Liu J (2016). Synthesis of BiOBr/WO_3_ p–n heterojunctions with enhanced visible light photocatalytic activity. CrystEngComm..

[56] Alam U, Verma N (2021). Direct Z-scheme-based novel cobalt nickel tungstate/graphitic carbon nitride composite: Enhanced photocatalytic degradation of organic pollutants and oxidation of benzyl alcohol. Colloids Surf. A Physicochem. Eng. Asp..

[57] Zhang XY (2022). Removal of U(VI) from aqueous solution via photocatalytic reduction over WO_3_/g-C_3_N_4_ composite under visible light. Chem. Eng. J..

[58] Huang L (2013). Synthesis and characterization of g-C_3_N_4_/MoO_3_ photocatalyst with improved visible-light photoactivity. Appl. Surf. Sci..

[59] Mersal M, Zedan AF, Mohamed GG, Hassan GK (2023). Fabrication of nitrogen doped TiO_2_/Fe_2_O_3_ nanostructures for photocatalytic oxidation of methanol based wastewater. Sci. Rep..

