# Supplementary material for: Enhanced Fenton-like process over Z-scheme MoO3 surface decorated with Fe2O3 under visible light
Source: Sci Rep. 2024 Apr 5;14:8007. doi: 10.1038/s41598-024-58634-2 (PMC10997789; doi:10.1038/s41598-024-58634-2)
Supplement: Supplementary file 1 — Supplementary Information. [file 41598_2024_58634_MOESM1_ESM.docx]

Supplementary Information

Enhanced Fenton-like Process over Z-scheme MoO_3_ Surface Decorated with Fe_2_O_3_ Under Visible Light

**Hsien-Tse Hsu^1^, Shao-Ying Lin^1^, Ya-Ting Lu^2^, Yao-Yuan Chuang*^1^, and Shiow-Huey Chuang*^1^**

1. Department of Applied Chemistry, National University of Kaohsiung, Kaohsiung 81148, Taiwan
2. Department of Chemical and Materials Engineering, National University of Kaohsiung, Kaohsiung 81148, Taiwan

*Corresponding authors: [shchuang@nuk.edu.tw](mailto:shchuang@nuk.edu.tw) (S.-H. Chuang), [ychuang@nuk.edu.tw](mailto:ychuang@nuk.edu.tw) (Y.-Y. Chuang)

(a) h-MO

**
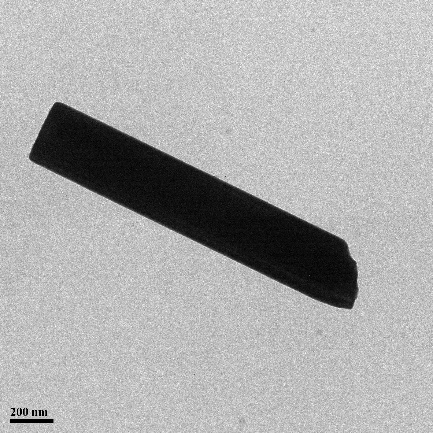

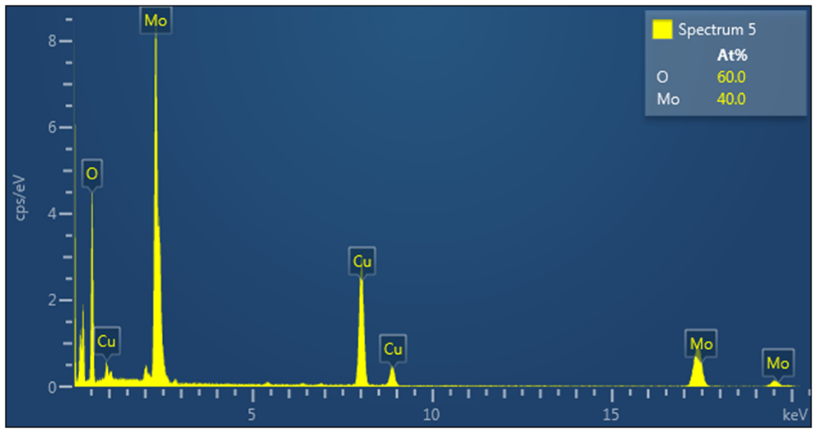
**

(b) h-MO-0

**
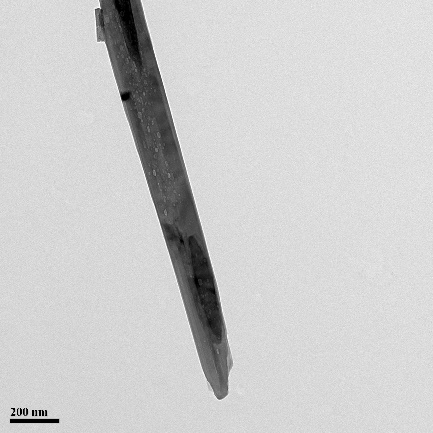
**
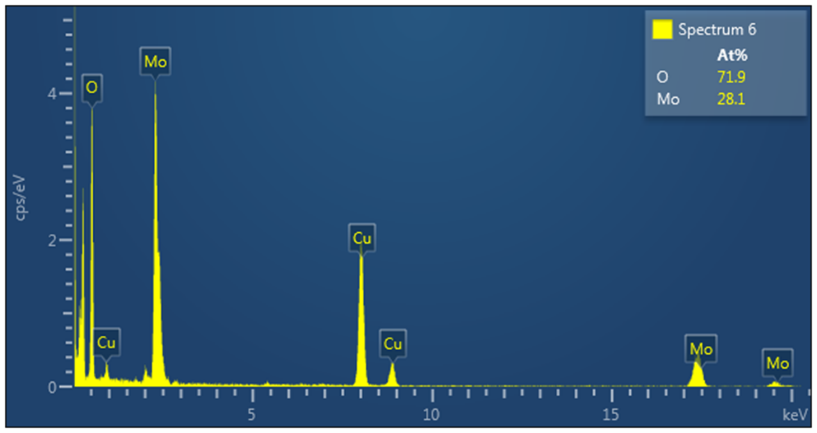


(c) h-MO-7

**
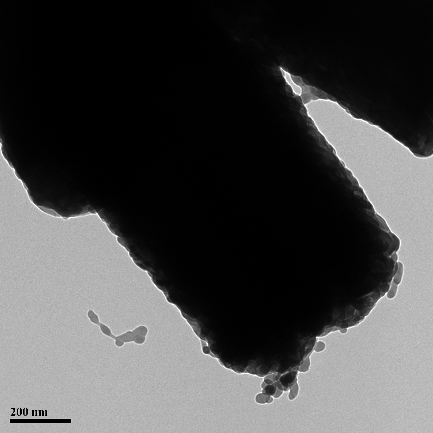
**
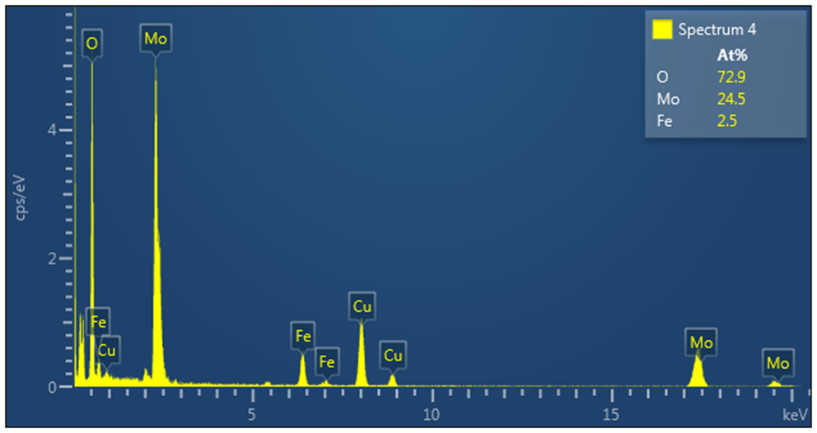


(d) h-MO-14


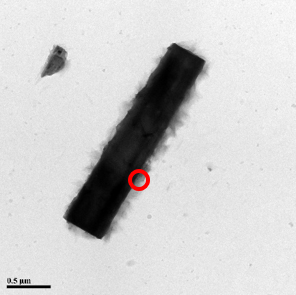

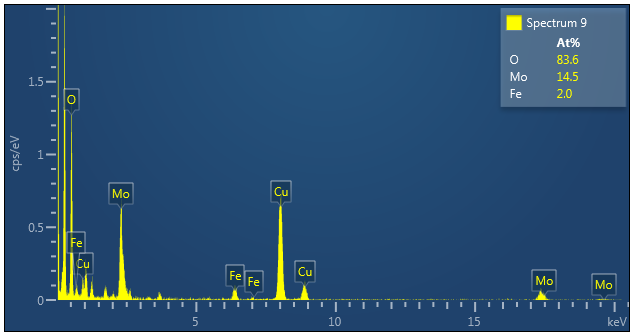

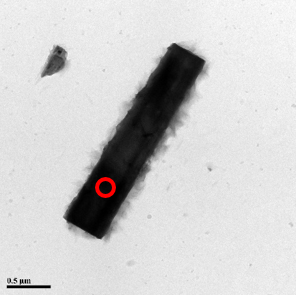

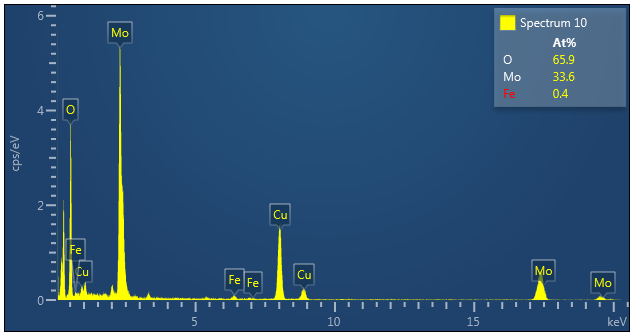


**Fig. S1** TEM and EDS compositional analysis of (a) h-MO, (b) h-MO-0, (c) h-MO-7, and (d) h-MO-14.


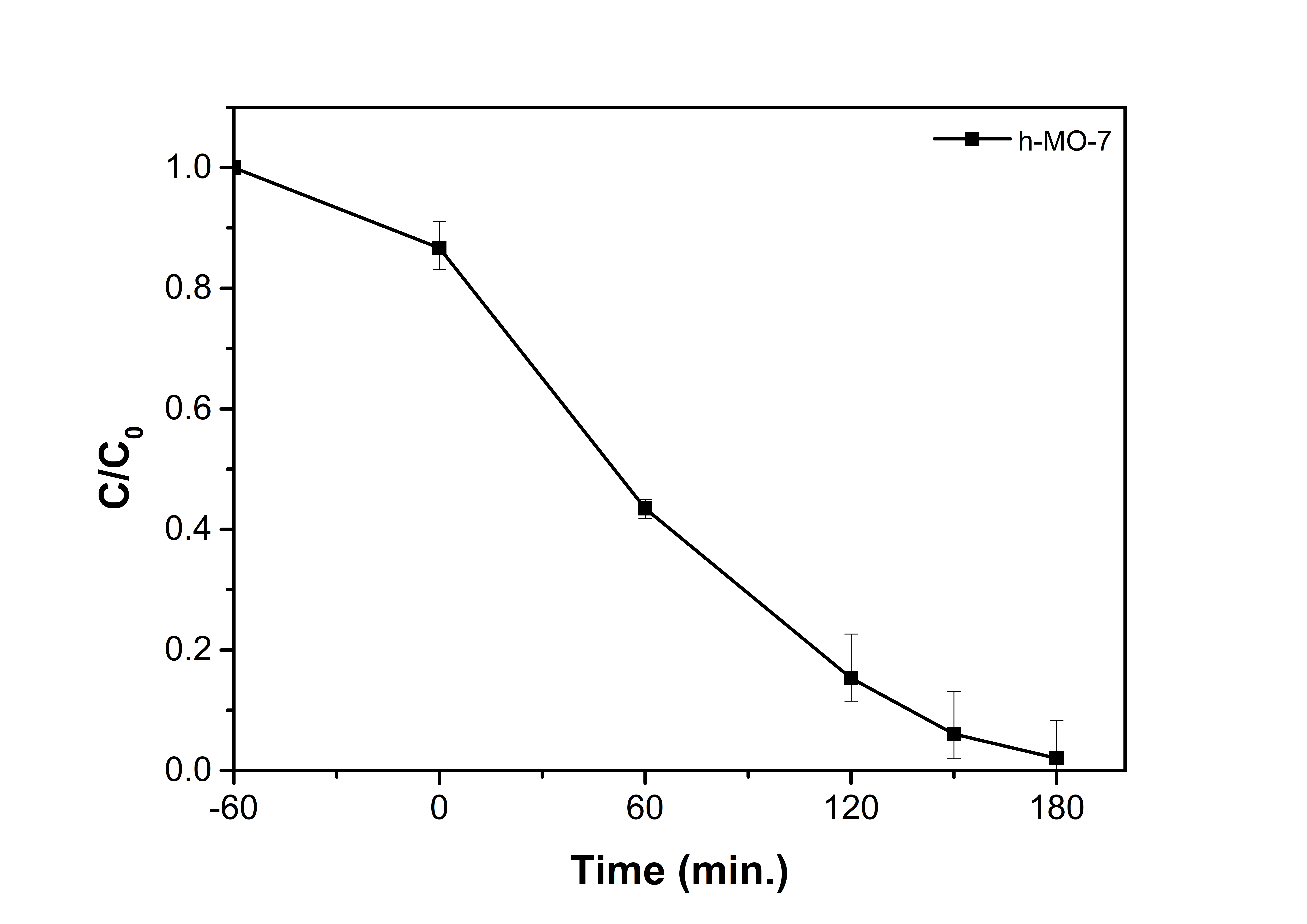


**Fig. S2** Photocatalytic degradation.

**Table S1** Comparison of organic pollutants degradation over different photocatalyst.

| **Photocatalyst** | **Pollutant** | **Photocatalyst / pollutant concentration** | **Time (min.)** | **Efficiency^*^ (mg/g / hr.)** | **Ref.** |
| --- | --- | --- | --- | --- | --- |
| *h*-MoO_3_ | MB | 50 mg / 100 mL of 10 mg L^-1^ | 180 | 6.47 | 14 |
| Fe_2_O_3_/MoO_3_ | TC | 20 mg / 40 mL of 20 mg L^-1^ | 90 | 21.6 | 23 |
| α-MoO_3_ | RhB | 60 mg / 60 mL of 10 mg L^-1^ | 240 | 2.45 | 31 |
| BiPO_4_/BiWO_6_ | RhB | 50 mg / 50 mL of 10 mg L^-1^ | 100 | 5.52 | 48 |
| Al_2_O_3_/BiPO_4_ | MB | 50 mg / 50 mL of 20 mg L^-1^ | 180 | 6.52 | 49 |
| Fe_2_O_3_/MoO_3_ | MB | 20 mg / 100 mL of 8 mg L^-1^ | 180 | 18.3 | This work |

*The efficiency is calculated from the data reported on the corresponding references.
